# Ion Channel Integration and Functional Coupling in Salivary Gland Fluid Secretion

**DOI:** 10.3390/cells15040369

**Published:** 2026-02-19

**Authors:** Tarek Mohamed Abd El-Aziz, Brij B. Singh

**Affiliations:** 1 Center for Regenerative Sciences, School of Dentistry, University of Texas Health Science Center San Antonio, San Antonio, TX 78229, USA; 2 Zoology Department, Faculty of Science, Minia University, El-Minia 61519, Egypt

**Keywords:** salivary glands, ion channels, fluid secretion, calcium signaling, epithelial transport

## Abstract

Salivary glands produce saliva through precisely coordinated epithelial ion transport processes. Ion channels are essential components of the molecular machinery that convert neural and hormonal signals into targeted ion and water flux. This review focuses on the integrated molecular and cellular mechanisms by which ion channels cooperate to generate salivary fluid under physiological conditions. Saliva formation proceeds through two sequential stages: isotonic primary fluid secretion by acinar cells, followed by ionic modification within the ductal epithelium. Parasympathetic stimulation activates muscarinic M1/3 receptors, initiating intracellular calcium signaling through inositol 1,4,5-trisphosphate-dependent release from the endoplasmic reticulum and sustained calcium entry via Orai1/TRPC channels. Elevated cytosolic calcium activates apical ANO1/TMEM16A chloride channels, the rate-limiting step in acinar fluid secretion, together with basolateral calcium-activated potassium channels that preserve the electrochemical driving force for chloride efflux. Chloride accumulation is maintained by Na^+^/K^+^-ATPase and the Na^+^-K^+^-2Cl^−^ cotransporter, while osmotic gradients drive water movement through apical aquaporin-5 and basolateral aquaporin-1/3. As primary saliva traverses the ductal system, epithelial sodium channels, CFTR, and additional ion transport pathways reabsorb sodium and chloride and secrete potassium and bicarbonate, producing hypotonic final saliva. By synthesizing calcium signaling, chloride and potassium conductance, sodium handling, and epithelial polarity into a unified framework, this review establishes ion channel integration as the fundamental basis of salivary gland fluid secretion.

## 1. Introduction

Salivary glands are essential exocrine organs that produce and secrete saliva, a complex biological fluid crucial for maintaining oral health, initiating digestion, and facilitating speech/swallowing and protecting the hard and soft tissues of the oral cavity. In humans, the salivary system contains three major pairs of glands, the parotid, submandibular, and sublingual glands, along with numerous minor glands distributed throughout the oral mucosa. Together, these glands produce 0.5 to 1.5 L of saliva daily under physiological conditions, reflecting the remarkable capacity of salivary epithelia to sustain high-volume fluid secretion [1,2]. The functional unit of salivary glands consists of acinar cells, which are responsible for primary fluid secretion, and ductal cells, which modify the ionic composition of saliva. Acinar cells are classified as serous, mucous, or seromucous based on the nature of their secretory products. Serous acini, predominant in the parotid glands, secrete a watery fluid rich in enzymes such as α-amylase, while mucous acini, found primarily in the sublingual glands, produce viscous mucin-rich secretions. The submandibular glands contain both cell types, producing mixed secretions [3,4]. In addition, the myoepithelial cells surround the acini and smaller ducts, contracting to facilitate saliva expulsion into the oral cavity.

Saliva performs multiple critical functions beyond lubrication. It contains antimicrobial proteins that provide the first line of defense against oral pathogens, digestive enzymes that initiate food breakdown, and buffering systems that stabilize oral pH and protect against dental caries [5]. In addition, saliva contains growth factors and proteins essential for tissue repair and the maintenance of oral mucosal integrity [6]. The loss or reduction in salivary function, known as xerostomia or dry mouth, significantly increases sensitivity to dental decay, oral infections, and difficulty in eating and speaking [7].

The production of saliva requires precise coordination among numerous ion channels, transporters, and water channels that are spatially organized across the polarized membranes of acinar and ductal epithelial cells. While the molecular identity, biophysical properties, and individual physiological roles of many of these transport proteins have been extensively characterized over the past several decades, considerably less attention has been devoted to understanding how these diverse components operate together as a coherent system. Salivary fluid secretion does not arise from the activity of any single channel in isolation but instead depends on coordinated interactions among multiple channel families, signaling pathways, and transport mechanisms distributed across distinct cellular and subcellular compartments. This review integrates current knowledge of ion channel function in salivary glands to highlight functional coupling and coordinated regulation as the fundamental organizing principles underlying salivary gland fluid secretion. To achieve this objective, the review first describes the two-stage model of acinar–ductal coordination that establishes the physiological framework for saliva formation. It then systematically examines the major ion channel families that execute this process: calcium channels that initiate and sustain the intracellular signaling cascades driving secretion, chloride channels that provide the rate-limiting conductance for fluid output, and potassium and sodium channels that maintain the electrochemical driving forces and mediate ductal salt reabsorption. This is followed by a discussion of aquaporins that couple ion gradients to transcellular water movement, cotransporters and exchangers that maintain the electrolyte gradients required for sustained transport, and the intracellular signaling pathways through which the autonomic nervous system regulates ion channel activity. Finally, these individual components are synthesized into a unified framework that identifies key organizing principles, including hierarchical rate-limiting steps, functional redundancy, non-canonical channel roles, and the critical importance of channel localization, and highlights unresolved questions for future investigation.

This review integrates findings from peer-reviewed publications that provide mechanistic, physiological, or translational insights into ion channel function in salivary gland secretion. The literature was selected based on the following criteria: (1) relevance to ion transport mechanisms in acinar or ductal epithelial cells of major salivary glands; (2) inclusion of experimental evidence from genetic, pharmacological, electrophysiological, or imaging-based approaches; and (3) publication in indexed journals with emphasis on studies from the past 20 years. Seminal older studies were included where foundational discoveries were necessary to establish context (e.g., identification of inositol trisphosphate signaling in salivary glands). Attention was given to articles employing knockout models, fluid secretion assays, and advanced imaging or structural techniques. Studies with direct clinical or translational relevance, including gene therapy, radiation injury, or autoimmune disease models (e.g., Sjögren’s syndrome), were prioritized to strengthen the therapeutic perspective of the review. Review articles were used selectively to provide broader context.

## 2. Acinar–Ductal Coordination of Epithelial Ion Transport in Saliva Fluid Formation

**Figure 1:**
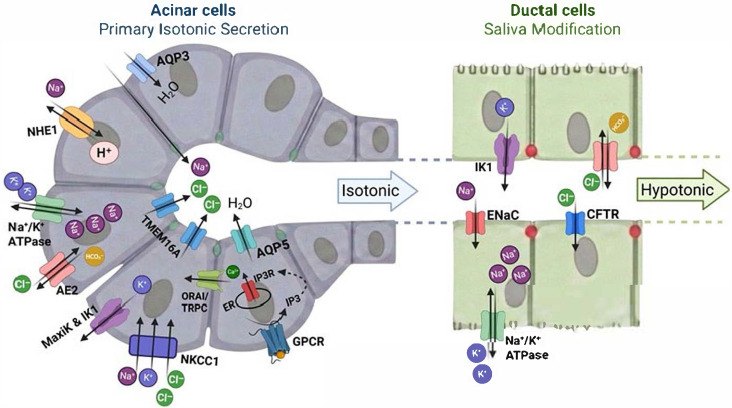
Mechanisms of primary isotonic saliva secretion by acinar cells and hypotonic saliva modification by ductal cells. Acinar cells generate primary isotonic saliva through coordinated basolateral Na^+^ and Cl^−^ uptake, apical Cl^−^ secretion, and transcellular water movement. As saliva passes through the ductal system, ductal epithelial cells reabsorb Na^+^ and Cl^−^ via apical transporters and channels, while limited water permeability results in the formation of hypotonic final saliva. Created in BioRender. Mohamed, T. (2026) https://BioRender.com/g0ktdf3.

Salivary secretion proceeds through a two-stage process requiring precise spatial organization of ion channels and transporters within polarized epithelial cells, coupled with temporal coordination of their activities in response to neural and hormonal stimuli [8,9]. Acinar cells exhibit distinct apical and basolateral membrane domains equipped with specific ion channels and transporters enabling directional ion transport. The apical membrane faces the acinar lumen and contains calcium-activated chloride channels (primarily TMEM16A) and aquaporin-5 (AQP5). The basolateral membrane expresses store-operated calcium channels (Orai1, TRPC1), calcium-activated potassium channels, the Na^+^/K^+^-ATPase, and the Na^+^-K^+^-2Cl^−^ cotransporter (Figure 1) [9].

Parasympathetic stimulation via acetylcholine activation of muscarinic M1/M3 receptors triggers phospholipase C-dependent production of inositol 1,4,5-trisphosphate (IP_3_) and diacylglycerol. IP_3_ binds to IP_3_ receptors on the endoplasmic reticulum, inducing Ca^2+^ release from intracellular stores [10,11]. The resulting elevation in cytosolic Ca^2+^ concentration activates apical Cl^−^ channels, driving Cl^−^ efflux into the acinar lumen. Simultaneously, Ca^2+^ activates basolateral potassium channels, causing K^+^ efflux that hyperpolarizes the membrane potential and maintains the electrochemical driving force for continued Cl^−^ secretion [12,13]. Chloride secretion creates a transepithelial electrical gradient and osmotic driving force promoting paracellular Na^+^ movement and transcellular water flux through AQP5, resulting in isotonic primary secretion [14]. The basolateral NKCC1 maintains intracellular Cl^−^ concentrations at 40–60 mM, well above the predicted equilibrium of approximately 10 mM, enabling sustained secretion [15,16]. Na^+^/K^+^-ATPase maintains the Na^+^ and K^+^ gradients driving NKCC1 activity, linking cellular energy metabolism to ion transport [17]. Sustained secretion requires prolonged calcium signaling supported by store-operated calcium entry (SOCE) through Orai1 and TRPC channels [18,19,20].

As isotonic primary fluid traverses the ductal system, it undergoes substantial ionic modification. Ductal cells reabsorb Na^+^ and Cl^−^ while secreting K^+^ and HCO_3_^−^, converting isotonic primary secretion into hypotonic final saliva [21]. Sodium concentration typically decreases from approximately 140 mM in primary saliva to 5–40 mM in final saliva, while K^+^ increases from approximately 5 mM to 15–30 mM. Ductal Na^+^ reabsorption occurs primarily through apical epithelial sodium channels (ENaC), with basolateral exit via Na^+^/K^+^-ATPase [22,23]. Chloride reabsorption proceeds transcellularly through apical CFTR and basolateral ClC-2 channels [24]. Potassium secretion into ductal fluid occurs through apical K^+^ channels, including calcium-activated potassium channels [25]. Bicarbonate secretion, mediated by CFTR, Cl^−^/HCO_3_^−^ exchangers (SLC26A6), and Na^+^/HCO_3_^−^ cotransporters, contributes to saliva’s buffering capacity essential for neutralizing dietary acids and maintaining oral pH homeostasis [26,27]. Ductal Ca^2+^ reabsorption is mediated by the apical calcium-sensing receptor (CaSR), which co-localizes with TRPC3 channels; CaSR activation triggers TRPC3-dependent Ca^2+^ entry, initiating transcellular Ca^2+^ flux proposed to protect against sialolithiasis [28,29]. The relative water impermeability of ductal epithelium, due to low aquaporin expression, prevents water reabsorption despite the osmotic gradient, maintaining the hypotonic character of final saliva [30,31]. Tight junctions between ductal epithelial cells restrict paracellular ion flux, resulting in high transepithelial resistance across the ductal epithelium [32].

These acinar and ductal transport mechanisms demonstrate that salivary fluid secretion arises from the coordinated activity of multiple ion channels, transporters, and water channels organized across polarized epithelial membranes. Secretory output is determined not only by the presence of individual transport pathways but also by their functional coupling, spatial segregation, and coordinated regulation in response to physiological stimulation. This integrated epithelial architecture enables directional ion movement, osmotic water flux, and the efficient modification of primary saliva as it traverses the ductal system. The following sections examine the major classes of ion channels and transport pathways that contribute to this coordinated process and define their specific roles in salivary gland physiology.

## 3. Calcium Channels Regulating Intracellular Signaling and Secretion

Calcium ions act as universal second messengers in salivary gland epithelial cells, mediating the transition from receptor activation to secretory output. The spatiotemporal dynamics of intracellular Ca^2+^ signals are determined by the coordinated activity of multiple channel types located on both the plasma membrane and intracellular organelles. These channels can be broadly categorized into SOCE channels, transient receptor potential (TRP) channels, voltage-dependent Ca^2+^ channels (VDCCs), and mechanosensitive ion channels. Each channel family contributes distinct properties to the calcium signaling toolkit, enabling salivary glands to respond appropriately to diverse physiological stimuli.

### 3.1. Mechanisms of Calcium Entry and Sustained Signaling in Salivary Gland Epithelium

SOCE represents the primary mechanism for sustained Ca^2+^ influx in salivary gland acinar cells following receptor-mediated depletion of endoplasmic reticulum (ER) Ca^2+^ stores. This pathway is mediated primarily by Orai1, the pore-forming subunit of the calcium release-activated calcium (CRAC) channel, which exhibits extremely high calcium selectivity and is activated through direct physical interaction with STIM1, the ER-resident Ca^2+^ sensor [33,34,35,36]. When ER Ca^2+^ levels decrease below approximately 400 μM, STIM1 undergoes conformational changes that promote oligomerization and translocation to ER–plasma membrane junctions, where it directly binds to Orai1 and induces channel opening [37,38]. Complementing Orai1, TRPC1 and TRPC3 channels contribute to calcium entry through distinct mechanisms. TRPC1 forms non-selective cation channels that may be recruited to STIM1-Orai1 clusters following store depletion, potentially contributing more significantly during weak or oscillatory stimulation patterns [39,40]. TRPC3, in contrast, is robustly activated by diacylglycerol (DAG) produced during PLC activation, enabling rapid calcium entry responses that complement the slower development of SOCE [41,42,43]. An emerging determinant of stimulus–secretion coupling is mitochondrial Ca^2+^ buffering, which shapes the amplitude and spatiotemporal pattern of cytosolic Ca^2+^ signals by rapidly taking up Ca^2+^ within microdomains generated near ER Ca^2+^ release sites [44]. In exocrine acinar cells, mitochondrial Ca^2+^ uptake has been shown to modulate Ca^2+^ oscillations and the spread of Ca^2+^ signals, thereby influencing downstream activation of Ca^2+^-dependent effectors [45]. In pathological contexts relevant to salivary glands, excessive mitochondrial Ca^2+^ loading can drive mitochondrial dysfunction and downstream loss of SOCE components, as demonstrated in the TRPM2-dependent radiation injury pathway [46]. In addition to STIM1, STIM2 contributes to SOCE by sensing smaller decreases in ER Ca^2+^ and facilitating Orai1 activation under weaker or more sustained stimulation conditions [47,48]. Mechanistically, STIM2 can enhance receptor-evoked SOCE by promoting STIM1 clustering at ER–plasma membrane junctions when ER Ca^2+^ depletion is modest, thereby increasing assembly of functional STIM1–Orai1 complexes [49]. Consistent with this role, salivary-gland STIM2 deletion reduces fluid secretion, particularly at relatively low stimulus intensities, supporting a distinct contribution of STIM2 to sustained/low-level signaling compared with STIM1 [49].

TRPM2 functions as a cellular sensor for reactive oxygen species (ROS) and plays a critical role in radiation-induced salivary gland dysfunction. Following radiation exposure, ROS production triggers poly(ADP-ribose) polymerase (PARP) activation and ADP-ribose (ADPR) generation, which activates TRPM2 and causes sustained Ca^2+^ influx leading to mitochondrial calcium overload, caspase-3 activation, and STIM1 cleavage [46,50,51,52,53]. TRPM2 knockout mice show significant protection against radiation-induced salivary dysfunction, recovering to approximately 60–70% of baseline secretion compared to only 30% in wild-type animals [50]. TRPV4, activated by hypotonic cell swelling and mechanical stimuli, mediates regulatory volume decrease (RVD) through functional coupling with AQP5 and contributes to muscarinic-stimulated secretion [54,55,56,57]. The thermosensitive channels TRPV1, TRPV3, and TRPM8 modulate salivation primarily through the activation of oral sensory nerves rather than direct effects on acinar cells, with TRPV1 agonists such as capsaicin stimulating salivary flow through reflex mechanisms [58,59,60,61,62,63].

Mechanosensitive Piezo1 channels represent a recently characterized pathway linking mechanical forces to calcium signaling in salivary glands. Piezo1 forms massive homotrimeric complexes containing 114 transmembrane domains that sense membrane tension and conduct Ca^2+^-permeable cation currents [64,65,66,67]. During development, Piezo1 is expressed specifically in acinar-forming epithelial cells at embryonic days 14–16, and siRNA-mediated knockdown significantly impair glandular morphogenesis [68]. In pathological contexts, PIEZO1 is upregulated during ionizing radiation-induced salivary gland hypofunction and correlates with inflammatory and fibrotic markers, suggesting mechanotransduction may contribute to radiation injury progression [69,70].

Voltage-dependent calcium channels, particularly L-type channels (CaV1.1, CaV1.2, CaV1.3), contribute primarily to salivary gland development rather than mature secretory function. These channels localize to peripheral cell layers of developing epithelial buds and regulate branching morphogenesis through MAPK/ERK signaling, with pharmacological inhibition by nifedipine causing dose-dependent impairment of epithelial bud formation and cleft initiation [71,72,73,74]. In mature salivary tissue, the functional significance of VDCCs remains less characterized, as acinar cells rely predominantly on store-operated mechanisms for secretion-coupled calcium entry.

The integrated function of these diverse calcium channels reflects evolutionary optimization that provides robust, sustained calcium signals capable of driving prolonged secretory responses while maintaining cellular calcium homeostasis (Table 1).

### 3.2. Intracellular Calcium Channels

While plasma membrane calcium channels mediate Ca^2+^ entry from the extracellular space, intracellular calcium release channels on the endoplasmic reticulum (ER) membrane are equally critical for generating the Ca^2+^ signals that drive salivary secretion. The ER serves as the principal intracellular Ca^2+^ store, maintaining luminal Ca^2+^ concentrations of 100–500 μM, which provides a substantial driving force for rapid Ca^2+^ release into the cytoplasm upon channel activation [94,95]. Two families of intracellular Ca^2+^ release channels reside on the ER membrane: inositol 1,4,5-trisphosphate receptors (IP_3_Rs) and ryanodine receptors (RyRs). The coordinated activity of these intracellular channels determines the spatial origin, temporal pattern, and amplitude of cytosolic Ca^2+^ signals, thereby shaping the secretory response to neural stimulation [8,96].

#### 3.2.1. IP3R—Inositol 1,4,5-Trisphosphate Receptors

IP_3_Rs are the primary intracellular Ca^2+^ release channels responsible for agonist-induced Ca^2+^ mobilization in salivary gland acinar cells. The discovery of IP_3_ as a second messenger originated from Berridge’s foundational research on blowfly salivary glands [94,97]. IP_3_Rs are large homotetrameric channel complexes containing an N-terminal IP_3_-binding core, a central regulatory domain, and a C-terminal channel domain with six transmembrane helices forming the ion-conducting pore [98,99]. Three mammalian isoforms exist (IP_3_R1, IP_3_R2, and IP_3_R3), with IP_3_R2 and IP_3_R3 being predominant in salivary acinar cells [100,101]. These receptors exhibit polarized distribution, concentrating in the apical region adjacent to secretory granules, ensuring that Ca^2+^ signals initiate at the luminal pole before transmitting basally as Ca^2+^ waves [96,102,103]. IP_3_R activation requires the simultaneous binding of IP_3_ and Ca^2+^. Cytosolic Ca^2+^ exhibits biphasic regulation: low concentrations (~100–300 nM) enhance channel opening through CICR, while elevated concentrations (>300 nM) inhibit activity, preventing Ca^2+^ overload [104]. This bell-shaped Ca^2+^ dependence underlies Ca^2+^ oscillations that optimize secretory responses [105,106].

Studies using IP_3_R2/IP_3_R3 double knockout mice demonstrated the complete elimination of agonist-induced Ca^2+^ signaling and fluid secretion, establishing the absolute requirement for these receptors in exocrine function [100]. The functional coupling between IP_3_Rs and SOCE is essential for sustained secretion. ER Ca^2+^ depletion following IP_3_R-mediated release triggers STIM1 oligomerization and Orai1 activation, initiating SOCE that refills depleted stores [107,108]. Clinical relevance has been demonstrated in Sjögren’s syndrome, where reduced IP_3_R2 and IP_3_R3 expression correlates with defective Ca^2+^ release even in morphologically intact acini [109].

#### 3.2.2. Ryr—Ryanodine Receptors

RyRs are large homotetrameric channel complexes (>2 megadaltons (MDa)) that complement IP_3_R function in salivary glands [110,111]. Although best characterized in striated muscle, functional RyRs in salivary acinar cells amplify Ca^2+^ signals through CICR [112,113]. Three isoforms exist (RyR1, RyR2, RyR3), with RyR3 being predominant in mouse parotid acinar cells [114].

Unlike apically concentrated IP_3_Rs, RyRs show broader distribution throughout the ER network, enabling amplification and propagation of Ca^2+^ signals from apical initiation sites toward basolateral regions [115]. RyR3 is the predominant isoform in parotid acinar cells and is activated by cADPR, with Ca^2+^ release inhibited by RyR antagonists (ruthenium red, ryanodine) but unaffected by the IP_3_R antagonist heparin [116]. Characteristically, salivary RyR3 channels are caffeine-insensitive, distinguishing them from RyR1 and RyR2 isoforms in excitable tissues. RyRs integrate with sympathetic signaling cyclic AMP generated by β-adrenergic activation, which enhances cADPR-induced Ca^2+^ release [113,116]. Calmodulin improves cADPR-induced release while inhibiting IP_3_-induced release, providing differential modulation [116]. This suggests that IP_3_Rs and RyRs occupy distinct Ca^2+^ pools, with RyRs contributing more to sympathetically mediated protein secretion while IP_3_Rs dominate parasympathetically driven fluid secretion [13,115].

## 4. Chloride Channels as Primary Drivers of Fluid Secretion

Chloride channels constitute the rate-limiting conductance for fluid secretion by salivary gland epithelia, establishing the osmotic gradient that drives transcellular water movement. The transepithelial flux of Cl^−^ ions provide the primary electrochemical driving force for both paracellular Na^+^ movement and osmotic water transport through aquaporins, positioning chloride channels as the central molecular determinants of salivary output [8,9]. Multiple chloride channel families with distinct activation mechanisms, subcellular localizations, and physiological roles contribute to salivary gland function in both acinar secretory cells and ductal modification processes.

In acinar cells, calcium-activated chloride channels (CaCCs), predominantly ANO1/TMEM16A, mediate the apical Cl^−^ efflux that initiates primary fluid secretion [12,117]. ANO1 transduces muscarinic receptor-induced Ca^2+^ signals into chloride conductance with remarkable efficiency, exhibiting Ca^2+^ sensitivity in the submicromolar to low micromolar range (EC_50_ ~ 0.3–1 µM) that matches physiological intracellular Ca^2+^ elevations during secretagogue stimulation [118]. Genetic deletion studies have unequivocally established ANO1 as the essential CaCC mediating salivary fluid secretion, with ANO1^−^/^−^ mice exhibiting complete loss of carbachol-stimulated Cl^−^ efflux despite preserved Ca^2+^ signaling [12]. The identification of ANO1 in 2008 resolved decades of investigation into CaCC molecular identity and distinguished the functional channel from earlier candidates including bestrophin family members, which proved dispensable for salivation in knockout models [12,117,119,120]. Recent cryo-EM structure-function studies resolved TMEM16A/ANO1 as a homodimer with two independent conduction pathways and defined intramembrane Ca^2+^-binding sites that gate pore-lining helix movements to enable anion permeation, providing a structural basis for its high Ca^2+^ sensitivity [121].

The cystic fibrosis transmembrane conductance regulator (CFTR) serves complementary functions in ductal epithelium, where it mediates Cl^−^ reabsorption and HCO_3_^−^ secretion rather than driving primary fluid output [122,123,124,125]. CFTR is a cAMP-activated, ATP-gated channel that forms functional complexes with scaffolding proteins including ezrin-radixin-moesin (ERM) proteins at luminal membranes of granular and reabsorptive duct cells [123]. Unlike the essential role of ANO1 in acinar secretion, genetic ablation of CFTR does not abolish stimulated saliva secretion, reflecting redundant chloride transport mechanisms in acinar cells [126,127]. However, CFTR mutations cause elevated Na^+^ and Cl^−^ concentrations in the saliva of cystic fibrosis patients, confirming its critical contribution to ductal salt reabsorption and bicarbonate secretion [128,129].

Voltage-gated chloride channels of the ClC family, particularly ClC-2 and ClC-3, have been investigated as potential contributors to salivary gland chloride transport [130,131,132]. ClC-2 exhibits preferential expression in ductal cells with basolateral localization consistent with a role in Cl^−^ uptake, yet Clcn2^−^/^−^ mice display normal saliva secretion, indicating functional redundancy with other transport mechanisms [131,133]. Similarly, ClC-3 proves dispensable for acinar secretion and volume regulation in knockout models, though its expression in peri-glandular vascular smooth muscle suggests potential indirect effects on glandular blood flow [132].

Volume-regulated anion channels (VRACs), comprising heterohexameric assemblies of leucine-rich repeat-containing 8 (LRRC8) proteins, maintain cell volume homeostasis during the dynamic osmotic changes accompanying secretion [134,135,136,137,138,139]. LRRC8A (SWELL1) serves as the obligatory pore-forming subunit, while LRRC8B-E accessory subunits modulate channel properties including permeability to organic osmolytes [136,137]. In salivary acinar cells, VRAC activation facilitates regulatory volume decrease (RVD) following hypotonic swelling by coordinating Cl^−^ efflux with K^+^ channel activation [140,141]. Beyond volume regulation, emerging evidence suggests that VRACs may function as conduits for ATP and other signaling molecules, potentially contributing to autocrine/paracrine regulation of secretion [142].

The integrated function of these chloride channel families reflects a hierarchical organization wherein ANO1 provides the essential, rate-limiting conductance for primary secretion, while CFTR, VRACs, and ClC channels contribute to ductal modification, volume homeostasis, and auxiliary transport functions (Table 2).

## 5. Potassium and Sodium Channels in Secretory and Absorptive Functions

Maintenance of ionic homeostasis across salivary gland epithelia requires the coordinated function of multiple potassium and sodium channels that work in concert with pumps, exchangers, and the chloride channels. While ANO1 provides the rate-limiting chloride conductance driving primary fluid secretion, potassium channels support equally essential roles by maintaining the electrochemical driving force for chloride efflux, enabling regulatory volume to decrease following cell swelling, and mediating potassium secretion into the luminal fluid [13,155,156]. In ductal cells, sodium channels facilitate the electrogenic reabsorption of sodium from primary saliva, contributing to the generation of hypotonic final saliva characteristic of mammalian salivary secretions [8,151,157].

Calcium-activated potassium channels constitute the principal K^+^ conductance pathway in salivary acinar cells, with two functionally distinct classes, large-conductance BK channels (KCa1.1) and intermediate-conductance IK channels (KCa3.1), operating in parallel to hyperpolarize the membrane during secretagogue stimulation [25,155,156,158]. BK channels, encoded by KCNMA1, exhibit the highest single-channel conductance among potassium channels and are distinguished by their dual activation mechanism requiring both membrane depolarization and elevated intracellular calcium [158,159,160]. Native parotid acinar BK channels display unusually high calcium sensitivity, with significant activity at 350–500 nM Ca^2+^, reflecting the expression of a parotid-specific splice variant [158,161]. IK channels (KCa3.1), encoded by KCNN4, are voltage-independent and activated solely by calcium binding to constitutively associated calmodulin, exhibiting half-maximal activation at approximately 350 nM calcium [161,162,163].

Remarkably, genetic studies reveal that BK and IK channels exhibit complete functional redundancy in supporting fluid secretion. Mice lacking either KCa1.1 or KCa3.1 alone maintain normal stimulated salivary secretion, demonstrating that either channel type suffices to preserve the electrochemical driving force [25,155]. However, double-knockout mice (KCa1.1^−^/^−^/KCa3.1^−^/^−^) exhibit severely impaired secretion reduced by 65%, establishing that potassium channel-mediated membrane hyperpolarization is essential for secretion [155]. This redundancy raises fundamental questions about the physiological rationale for expressing two seemingly interchangeable channels. Evidence suggests complex functional interactions between BK and IK, including membrane-delimited inhibition of BK channels by IK activation through a mechanism involving physical proximity and potential direct pore block by the IK N-terminus [164,165]. BK channels contribute to ductal potassium secretion. KCa1.1 localizes to apical membranes of striated and excretory duct cells, and the flow rate-dependent increase in salivary K+ concentration observed in wild-type mice is nearly eliminated in KCa1.1^−^/^−^ animals [166]. BK channels also participate in regulatory volume decrease following hypotonic cell swelling, with KCa1.1^−^/^−^ cells exhibiting substantially impaired RVD [158].

Inward rectifier potassium channels (Kir) exhibit species-specific expression patterns in salivary glands. Kir2.1 is prominently expressed in bovine parotid acinar cells, where it contributes to resting membrane potential stabilization, but appears absent in rodent salivary acinar cells [167]. This interspecies difference may relate to the unique physiology of ruminant salivary glands, which are secreted continuously without neural stimulation. The hypothesis that Kir channels specifically support spontaneous secretion is supported by observations that inward rectifier currents are absent from parotid acinar cells of unweaned lambs, which do not exhibit spontaneous secretion [167].

The epithelial sodium channel (ENaC) mediates the rate-limiting step for sodium reabsorption in salivary gland ducts. ENaC assembles as a heterotrimer of α, β, and γ subunits and localizes to apical membranes of duct cells [8,151,168,169,170]. This sodium reabsorption is critical for generating hypotonic final saliva, as ductal epithelium exhibits low water permeability, allowing salt reabsorption without equivalent water movement [8,13,171]. ENaC blockades with amiloride significantly impair Na+ reabsorption and abolish transepithelial potential difference in perfused gland preparations [151,157,171]. ENaC activity is regulated by glucocorticoids and mineralocorticoids through transcriptional and post-translational mechanisms involving SGK1 and appears functionally coupled to CFTR for coordinated Na+ and Cl^−^ reabsorption [151,168,169,172].

The integrated function of potassium and sodium channels reflects compartmentalized roles: basolateral K^+^ channels in acinar cells maintain driving force for secretion, apical K^+^ channels in ducts mediate K^+^ secretion, and apical ENaC in ducts enables Na+ reabsorption for saliva modification (Table 3).

## 6. Aquaporins and the Coupling of Ion and Water Transport

Water transport across salivary gland epithelia is fundamentally coupled with ion transport, as water moves passively along osmotic gradients generated by transepithelial ion flux. The molecular basis for rapid transcellular water movement remained unclear until the discovery of aquaporins (AQPs) in the early 1990s [14,30,183]. Aquaporins are integral membrane proteins that form selective water channels, facilitating osmotically driven water transport across cell membranes at rates far exceeding simple diffusion through lipid bilayers [30,31]. Salivary glands express multiple aquaporin isoforms with distinct subcellular localizations and functional properties [184,185,186].

Aquaporin-5 (AQP5) represents the predominant water channel mediating transcellular fluid secretion in salivary gland acinar cells. AQP5 exhibits highly polarized localization to apical membranes of acinar cells in parotid, submandibular, sublingual, and labial glands [30,31,183,186]. The functional significance of AQP5 was definitively established through AQP5 knockout mice, which exhibited a 60% reduction in pilocarpine-stimulated parotid saliva volume despite normal gland morphology and preserved expression of other secretory components [14]. The residual 40% secretion indicates that alternative water transport pathways can partially compensate, contrasting with the complete ablation observed following ANO1 deletion and reflecting ANO1’s role as the rate-limiting conductance [12,14]. AQP5 also participates in cell volume regulation through functional coupling with the mechanosensitive channel TRPV4. Following hypotonic cell swelling, water influx through AQP5 rapidly increases cell volume, activating TRPV4 to trigger calcium influx and subsequent volume-regulatory ion efflux. Cells lacking either AQP5 or TRPV4 exhibit impaired regulatory volume decrease, demonstrating bidirectional functional coupling [56]. Pathophysiological dysregulation of AQP5 is a central feature of both Sjögren’s syndrome and radiation-induced xerostomia. In Sjögren’s syndrome, AQP5 undergoes striking mislocalization from apical to basolateral membranes, effectively inverting the normal polarity required for secretion [185,186,187]. This redistribution occurs even in morphologically intact acini, suggesting that AQP5 dysfunction occurs independently of lymphocytic infiltration. Following radiation exposure, AQP5 expression decreases through transcriptional downregulation and increased protein degradation mediated by oxidative stress, while radiation also disrupts trafficking and membrane localization through cytoskeletal damage and loss of ezrin scaffolding [184,188,189,190].

Aquaporin-3 (AQP3) functions as the principal basolateral water channel in salivary acinar cells, enabling water uptake from the interstitial space [31,191]. Unlike the orthodox aquaporin AQP5 exhibiting high water selectivity, AQP3 is an aquaglyceroporin that facilitates the transport of water, glycerol, and small uncharged solutes including urea [192,193]. During stimulated secretion, acinar cells secrete fluid at rates approaching their own cell volume per minute, necessitating extremely rapid basolateral water influx. Simple diffusion through lipid bilayers would be insufficient; AQP3 increases basolateral membrane water permeability by 10- to 20-fold, enabling rapid water uptake matching apical secretion rates [191,194].

Aquaporin-1 (AQP1) is expressed in myoepithelial cells surrounding acini and intercalated ducts, as well as in vascular endothelium [31,195,196,197]. The functional significance of AQP1 in myoepithelial cells remains incompletely understood, though one hypothesis proposes that rapid water permeability enables volume homeostasis during contraction [197]. Importantly, AQP1 is absent or minimally expressed in normal salivary acinar and ductal epithelia, providing the conceptual foundation for AQP1-based gene therapy for radiation-induced xerostomia. Clinical trials of adenoviral-mediated AQP1 gene transfer to irradiated parotid glands demonstrated proof-of-concept efficacy, with treated patients showing objective increases in stimulated salivary flow and durable transgene expression at 3 years post-treatment [198,199,200].

Additional aquaporins detected in salivary glands include AQP4, which shows minimal acinar expression but may localize to ductal epithelia and myoepithelial cells, and AQP8, an intracellular aquaporin predominantly localized to mitochondrial inner membranes [31,188,191,201,202]. The functional contributions of these minor isoforms to salivary gland physiology remain to be fully characterized.

The integrated function of aquaporins in salivary secretion reflects polarized expression enabling vectorial water transport: basolateral AQP3 mediates water entry, apical AQP5 mediates water exit, and the osmotic gradient generated by ion channels and transporters provides the driving force (Table 4).

## 7. Cotransporter and Exchanger Systems Maintaining Electrolyte Balance in Salivary Glands

The generation of saliva requires the coordinated activity of multiple membrane transport proteins that collectively establish the electrochemical gradients necessary for transcellular ion movement. According to the two-stage hypothesis of salivary secretion, acinar cells produce a primary isotonic fluid rich in NaCl, which is subsequently modified by reabsorbing Na^+^ and Cl^−^ while secreting K^+^ and HCO_3_^−^, yielding hypotonic final saliva [13]. This complex process depends upon the functional integration of primary active transporters, secondary active cotransporters, and electroneutral exchangers distributed across the basolateral and apical membranes of both acinar and ductal epithelial cells.

Na^+^/K^+^-ATPase constitutes the primary active transporter that drives the entire secondary transport processes in salivary glands. This heterodimeric enzyme catalyzes the ATP-dependent exchange of three intracellular Na^+^ ions for two extracellular K^+^ ions, generating the steep transmembrane Na^+^ gradient (approximately 140 mM extracellular versus 10–20 mM intracellular) that drives all secondary active transport processes [21]. The electrogenic 3Na^+^:2K^+^ exchange stoichiometry contributes directly to the resting membrane potential and, together with K^+^ channels, establishes the negative intracellular voltage essential for Cl^−^ secretion. The cardiac glycoside ouabain demonstrates the essential role of this pump, with ouabain causing a 95.8% reduction in acetylcholine-stimulated saliva volume in isolated perfused rat submandibular glands [208].

The Na^+^-K^+^-2Cl^−^ cotransporter isoform 1 (NKCC1) represents the principal basolateral Cl^−^ uptake mechanism in salivary gland acinar cells, harnessing the inwardly directed Na^+^ gradient to accumulate intracellular Cl^−^ to concentrations approximately fivefold above the electrochemical equilibrium [21]. NKCC1 mediates the electroneutral cotransport of one Na^+^, one K^+^, and two Cl^−^ ions, functioning as part of a “Cl^−^ pump” mechanism that provides the driving force for apical Cl^−^ efflux through CaCCs [156]. The critical importance of NKCC1 was established through knockout studies demonstrating a >60% reduction in muscarinic-stimulated saliva volume in NKCC1-deficient mice [15]. Human patients with complete NKCC1 deficiency exhibit severe xerostomia alongside deafness and CFTR-like secretory defects, underscoring the non-redundant role of this cotransporter [209,210].

Na^+^/H^+^ exchangers (NHEs) defend intracellular pH against acidification while contributing to cell volume regulation and providing a secondary pathway for Cl^−^ uptake when coupled with Cl^−^/HCO_3_^−^ exchangers. NHE1 is the dominant functional isoform in acinar cells, with NHE1-deficient mice exhibiting complete failure of pH recovery from acid loads [211]. In ductal cells, NHE3 localizes to apical membranes and mediates luminal H^+^ secretion as part of the HCO_3_^−^ salvage mechanism [212]. The functional coupling between basolateral NHE1 and Cl^−^/HCO_3_^−^ exchangers provides approximately 30% of basolateral Cl^−^ loading during muscarinic stimulation [21].

Sodium bicarbonate cotransporters (NBCs) mediate coupled Na^+^ and HCO_3_^−^ transport, playing crucial roles in intracellular pH regulation and transepithelial HCO_3_^−^ secretion. The electrogenic NBCe1-B (SLC4A4) is the principal isoform in salivary glands, localizing to basolateral membranes of parotid acinar cells where it supports pH regulation and provides the HCO_3_^−^ substrate for apical secretion [213,214]. Gland-specific differences in NBC distribution exist, with submandibular duct cells showing a gradient from basolateral to apical localization along the ductal tree [214,215].

Cl^−^/HCO_3_^−^ exchangers participate in both basolateral Cl^−^ uptake and apical HCO_3_^−^ secretion. Two gene families encode these exchangers: the SLC4 family (Ae2, Ae4) and the SLC26 family (SLC26A6). Ae4 has begun as the critical exchanger for salivary secretion, with Ae4^−^/^−^ mice exhibiting 35% reduction in salivation [216]. Although AE2 is abundantly expressed, it appears to function mainly as a baseline ‘housekeeping’ Cl^−^/HCO_3_^−^ exchanger for pH and Cl^−^ homeostasis, whereas stimulated salivary secretion relies more on dynamically regulated pathways such as cAMP-activated AE4, making AE2 largely redundant in knockout secretion assays [216]. Ae4 is specifically activated by cAMP/PKA signaling, explaining its role in β-adrenergic-stimulated secretion [217]. In ductal cells, SLC26A6 mediates apical Cl^−^/HCO_3_^−^ exchange in the secretory direction, coupling with CFTR for HCO3^−^ secretion [21].

The integrated function of these transporters and exchangers reflects a hierarchical organization wherein Na^+^/K^+^-ATPase provides the primary driving force, NKCC1 mediates the bulk of Cl^−^ accumulation, and NHE/Cl^−^-HCO_3_^−^ exchanger coupling provides auxiliary Cl^−^ uptake while maintaining pH homeostasis (Table 5).

## 8. Intracellular Signaling Pathways Regulating Ion Channel Activity

**Figure 2:**
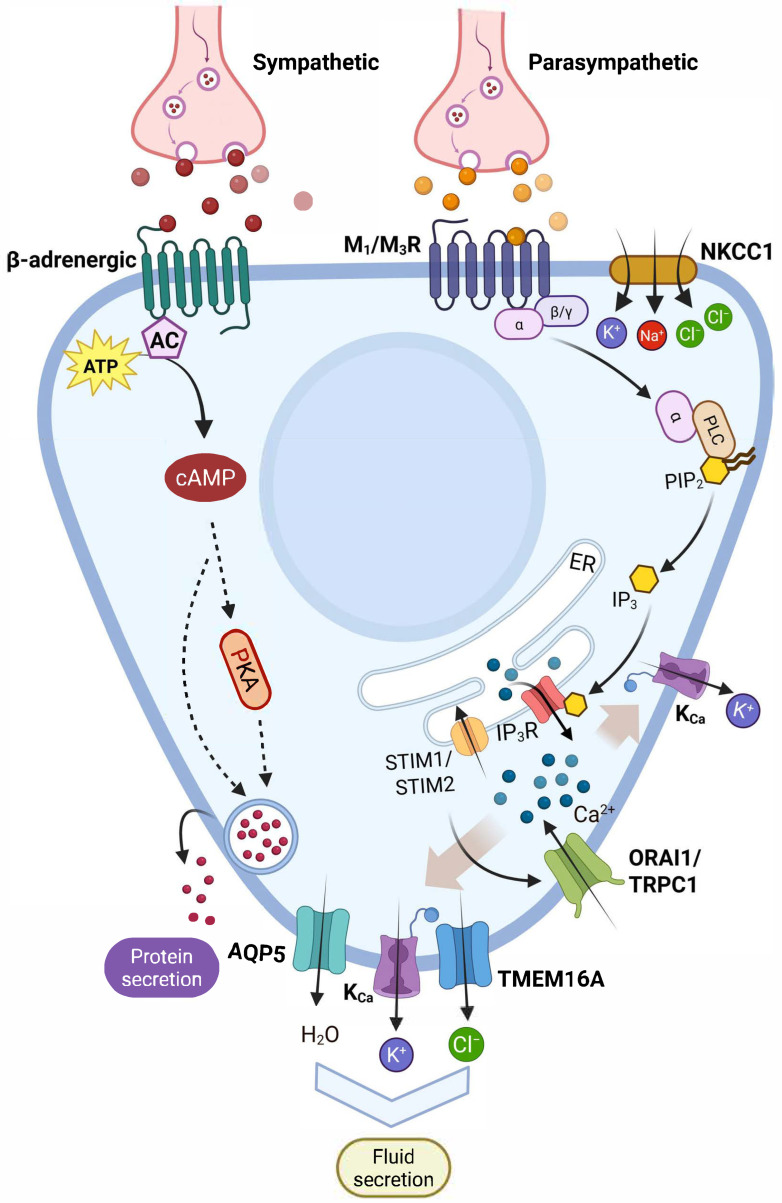
Intracellular signaling pathways regulating ion transport and secretion in salivary gland acinar cells. Parasympathetic and sympathetic inputs activate muscarinic (M1/M3) and β-adrenergic receptors, respectively, leading to PLC/IP_3_-mediated Ca^2+^ signaling and cAMP/PKA pathways. These signals coordinate Ca^2+^ entry, ion channel activity, water movement, and regulated protein and fluid secretion. AC: Adenylyl cyclase; cAMP: Cyclic adenosine monophosphate; ER: Endoplasmic reticulum; IP_3_: Inositol 1,4,5-trisphosphate; IP_3_R: Inositol 1,4,5-trisphosphate receptor; KCa: Ca^2+^-activated potassium channel; M1/M3R: Muscarinic acetylcholine receptor subtypes M1 and M3; PIP_2_: Phosphatidylinositol 4,5-bisphosphate; PKA: Protein kinase A; PLC: Phospholipase C. Created in BioRender. Mohamed, T. (2026) https://BioRender.com/jmt21ae.

Salivary gland function is organized by the autonomic nervous system through the coordinated activation of multiple signaling cascades that regulate ion channel activity, fluid secretion, and protein exocytosis [8,9]. The parasympathetic and sympathetic branches of the autonomic nervous system differentially control these processes through distinct yet interconnected molecular pathways (Figure 2) [8,9,20].

### 8.1. Parasympathetic Signaling Through Muscarinic Receptors

Parasympathetic stimulation constitutes the primary physiological trigger for salivary fluid secretion [225,226]. Acetylcholine released from parasympathetic nerve terminals activates muscarinic acetylcholine receptors (mAChRs) expressed in the basolateral membrane of acinar and ductal cells [226,227]. Of the five mAChR subtypes (M1–M5), salivary glands predominantly express M1 and M3 receptors, both of which couple to Gq/11 proteins [228,229].

Genetic evidence definitively establishes M3 as the primary mediator of physiological salivation. Mice lacking M3 receptors (M3^−^/^−^) exhibit profound deficits in carbachol-stimulated Ca^2+^ signaling and the complete absence of pilocarpine-induced fluid secretion [225,230]. These animals display severe phenotypes, including difficulty consuming dry food and postweaning growth impairment that is rescued by provision of a softened diet [225]. In contrast, M1^−^/^−^ mice show only modest reductions in Ca^2+^ responses, and carbachol-stimulated secretion requires higher agonist doses but remains functional [225,230]. Mice lacking both M1 and M3 receptors (M1/M3^−^/^−^) exhibit a complete loss of cholinergic Ca^2+^ mobilization, confirming that these two subtypes mediate the entirety of muscarinic-dependent Ca^2+^ signaling in acinar cells [225,230].

The canonical M3 signaling pathway proceeds through well-defined steps [18,231,232]: (1) Acetylcholine binding to M3 receptors induces GDP-GTP exchange on Gαq/11 subunits; (2) Activated Gαq/11 dissociates from Gβγ and binds phospholipase Cβ (PLCβ), particularly the PLCβ3 isoform enriched in salivary tissue [233]; (3) PLCβ hydrolyzes phosphatidylinositol 4,5-bisphosphate (PIP_2_) in the plasma membrane, generating two second messengers: inositol 1,4,5-trisphosphate (IP_3_) and diacylglycerol (DAG) [234]; (4) IP_3_ diffuses through the cytoplasm and binds to IP_3_ receptors (primarily IP_3_R2 and IP_3_R3 isoforms) on the endoplasmic reticulum, triggering Ca^2+^ release from intracellular stores [100,235]; (5) Store depletion activates store-operated Ca^2+^ entry (SOCE) through Orai1 and TRPC channels, producing a sustained elevation of cytosolic Ca^2+^.

The spatiotemporal pattern of Ca^2+^ signals encode specific functional outcomes. Muscarinic stimulation initially produces a rapid Ca^2+^ spike originating from IP_3_-mediated store release, followed by Ca^2+^ oscillations whose frequency increases with agonist concentration [236,237]. These Ca^2+^ signals activate multiple downstream effectors: (1) Apical ANO1/TMEM16A chloride channels open in response to submicromolar Ca^2+^, driving Cl^−^ efflux and fluid secretion [12]; (2) Basolateral IK_1_ (KCa3.1) potassium channels activate with EC_50_ ~300 nM Ca^2+^, maintaining the electrochemical gradient for continued Cl^−^ secretion [25]; (3) Aquaporin-5 water channels facilitate osmotic water movement across the apical membrane [183].

DAG, the other product of PIP_2_ hydrolysis, activates conventional protein kinase C (cPKC) isoforms, particularly PKC-α, in a Ca^2+^-dependent manner [234,238]. The synergistic action of DAG and elevated Ca^2+^ is critical for the sustained exocytosis of secretory granules. Studies in sublingual mucous cells demonstrate that initial transient Ca^2+^ release triggers a fusion-competent pool of granules, while PKC activation is required for the sustained phase of mucin secretion lasting beyond 5 min [238]. PKC-α translocates to the luminal borders of mucous cells during prolonged carbachol stimulation, consistent with its role in granule docking and priming [238].

### 8.2. Sympathetic Signaling Through Adrenergic Receptors

Sympathetic nerve terminals release norepinephrine (noradrenaline), which activates α-adrenergic and β-adrenergic receptors with distinct functional outcomes [8,9,20]. While parasympathetic stimulation primarily drives fluid secretion, sympathetic activation predominantly regulates protein secretion and exocytosis, though significant crosstalk exists between these pathways [239,240].

β-Adrenergic receptors couple to stimulatory G proteins (Gαs) that activate adenylyl cyclase, elevating intracellular cAMP levels and subsequently activating cAMP-dependent protein kinase A (PKA) [240,241]. Salivary glands express both β1 and β2 receptor subtypes, with β1 receptors mediating most isoproterenol-stimulated responses [242,243]. In rodent parotid glands, β-adrenergic stimulation produces robust protein secretion, primarily amylase, with 50-fold increases over baseline following maximal receptor activation [242,244].

The cAMP-PKA pathway regulates protein secretion through multiple mechanisms [244]: (1) PKA phosphorylates proteins involved in secretory granule trafficking, docking, and fusion with the apical membrane [244]; (2) PKA activates transcription factors including CREB (cAMP response element-binding protein), which regulates the expression of secretory proteins [245]; (3) PKA modulates ion channel activity, though this represents a minor component compared to the dominant role of Ca^2+^ in fluid secretion [10,240]. Type II PKA (PK-AII) appears to be the predominant cAMP receptor mediating secretion in parotid glands, as demonstrated by the synergistic stimulation of amylase secretion by cAMP analogs that selectively activate PK-AII regulatory and catalytic subunits [244]. This isoform-specific signaling may enable the selective modulation of β-adrenergic responses.

Beyond canonical cAMP-PKA signaling, β-adrenergic receptors activate exchange proteins directly activated by cAMP (Epac), which are guanine nucleotide exchange factors for the small GTPase Rap [10,239]. Epac mediates certain β-adrenergic effects independent of PKA, although its specific contributions to salivary secretion require further investigation [10,239].

α1-Adrenergic receptors couple to Gαq proteins and activate PLCβ, generating IP_3_ and DAG in a pathway parallel to muscarinic signaling [246,247]. Accordingly, α1 stimulation produces Ca^2+^ mobilization and can contribute to fluid secretion, particularly in submandibular glands [246,247]. α2-adrenergic receptors couple to inhibitory Gαi proteins and suppress salivary secretion by inhibiting responses to muscarinic, α1-adrenergic, and substance P stimulation [247]. This α2-mediated inhibition does not affect β-adrenergic responses, suggesting compartmentalized regulation of secretory pathways [247].

Importantly, extensive crosstalk occurs between Ca^2+^ and cAMP signaling systems [10,239,240]. PKA phosphorylates IP_3_ receptors at Ser1756, enhancing their Ca^2+^ sensitivity and increasing Ca^2+^ release in response to submaximal muscarinic stimulation [10,248]. This mechanism explains the synergistic effect of combined parasympathetic and sympathetic stimulation on fluid secretion observed in vivo [10,240]. In contrast, cAMP elevation inhibits muscarinic-stimulated ERK phosphorylation through PKA-dependent mechanisms, representing negative crosstalk that may limit certain signaling outputs [240,249].

### 8.3. Additional Regulatory Pathways

Beyond the canonical autonomic pathways, multiple additional signaling mechanisms fine-tune salivary gland ion channel activity and secretory function.

#### 8.3.1. Purinergic Signaling

ATP released through exocytosis or pannexin channels acts as an autocrine/paracrine signal via P2X and P2Y purinergic receptors [250,251]. P2X receptors are ligand-gated Ca^2+^-permeable channels that produce rapid Ca^2+^ influx [251,252]. P2X4 and P2X7 receptor activation is potentiated by β-adrenergic-induced cAMP elevation, representing another mode of sympathetic-purinergic crosstalk [251]. Muscarinic stimulation triggers exocytosis-dependent ATP release that secondarily activates P2X7 receptors, amplifying Ca^2+^ signals [251]. This purinergic signaling likely contributes to dynamic modulation of secretion in response to varying physiological demands [251].

#### 8.3.2. MAPK/ERK Signaling

Extracellular signal-regulated kinase (ERK) activation occurs downstream of both muscarinic and β-adrenergic receptors through distinct mechanisms [249,253]. Muscarinic receptors activate ERK through PKC-dependent pathways, while β-adrenergic receptors activate ERK through cAMP-dependent mechanisms that may involve Epac and/or transactivation of EGF receptors [249,253]. ERK phosphorylation regulates gene expression and cellular proliferation, contributing to adaptive responses and gland development [253].

#### 8.3.3. Calcium-Calmodulin-Dependent Kinases

Ca^2+^-calmodulin complexes activate CaMKII and other calcium-dependent kinases that phosphorylate ion channels and regulatory proteins [254]. While earlier studies suggested CaMKII involvement in chloride channel activation, genetic evidence demonstrates that ANO1 activation by Ca^2+^ does not require calmodulin or CaMKII [118]. Nevertheless, CaMKII likely regulates other aspects of secretory function including gene expression and metabolic adaptation [254].

#### 8.3.4. Nitric Oxide Signaling

Neuronal nitric oxide synthase (nNOS) is expressed in parasympathetic nerve terminals innervating salivary glands, and NO modulates both blood flow and epithelial secretion [255]. NO activates soluble guanylyl cyclase to produce cGMP, which activates cGMP-dependent protein kinase (PKG). This pathway regulates vascular tone in glandular blood vessels and may modulate ion channel activity, though the mechanisms remain incompletely defined [255,256].

#### 8.3.5. IRBIT and WNK1 Regulation

The IP_3_ receptor-binding protein released with IP_3_ (IRBIT) acts as a critical integrator of Ca^2+^ and cAMP signaling in secretory epithelia [27]. IRBIT is displaced from IP_3_ receptors upon IP_3_ binding and translocate to the plasma membrane where it scaffolds ion transporters including NBC (Na^+^-bicarbonate cotransporter) and CFTR [27]. IRBIT also modulates WNK1 kinase signaling, which regulates CFTR bicarbonate permeability [257]. This pathway is particularly important in pancreatic and salivary ductal cells for bicarbonate secretion [27,257].

The integration of this multiple signaling pathways enables salivary glands to precisely match secretory output to physiological demands while maintaining the appropriate ionic composition of saliva through the coordinated regulation of channels, transporters, and water permeability [8,9,21].

## 9. Translational Significance, Current Limitations, and Future Directions

**Figure 3:**
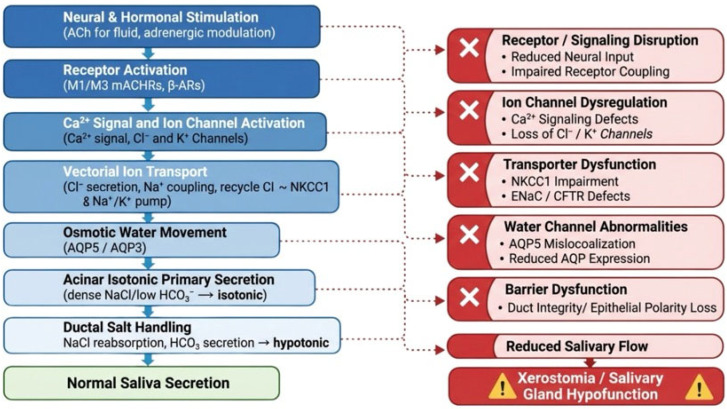
Integrated ion-water transport cycle in salivary fluid secretion and points of dysfunction. Normal secretion steps are shown in blue (**left**): neural/hormonal stimulation activates receptors to drive Ca^2+^-dependent channel activation, vectorial ion transport, osmotic water movement, isotonic acinar primary secretion, and ductal salt handling that generates hypotonic final saliva, resulting in normal saliva secretion (green). Disruptions at these stages (red, **right**), including receptor/signaling defects, ion channel dysregulation, transporter impairment, water channel abnormalities, or barrier dysfunction, reduce salivary flow and lead to xerostomia/salivary gland hypofunction.

The integrated understanding of ion channel function in salivary glands presented in this review carries substantial translational value for both basic research and clinical medicine (Figure 3). Xerostomia affects millions of individuals worldwide, arising from radiation therapy for head and neck cancers, autoimmune destruction in Sjögren’s syndrome, aging, and adverse effects of more than 500 commonly prescribed medications, yet effective treatments remain limited [7,12,24]. The identification of ANO1/TMEM16A as the indispensable, rate-limiting chloride conductance for muscarinic-dependent acinar fluid secretion positions this channel as a high-priority therapeutic target. Catalán et al. demonstrated that acinar-specific conditional deletion of Tmem16A in adult mice completely abolishes calcium-dependent salivation while preserving β-adrenergic-stimulated secretion, revealing an alternative secretory pathway whose molecular identity remains undefined and may represent an additional therapeutic opportunity [24]. Furthermore, the development of small-molecule TMEM16A activators that directly stimulate chloride conductance independently of upstream calcium signaling offers a pharmacological strategy to bypass defective signaling components in diseased glands [120]. The recognition that TRPM2 mediates radiation-induced salivary gland damage through an oxidative stress-dependent pathway involving poly(ADP-ribose) polymerase activation, sustained calcium influx, mitochondrial calcium overload, caspase-3 activation, and STIM1 cleavage provides a defined molecular cascade amenable to pharmacological intervention [46,50]. TRPM2 knockout mice demonstrated a >60% recovery of salivary function by 30 days post-irradiation compared with irreversible loss in wild-type animals, and treatment with the mitochondria-targeted antioxidant MitoTEMPO resulted in the near-complete protection of salivary gland secretion following both single and fractionated radiation doses [46,50,258]. The successful proof-of-concept Phase I clinical trial of adenoviral-mediated AQP1 gene transfer to irradiated parotid glands further illustrates how mechanistic knowledge of ion channel function can be translated into therapeutic strategies, with objective increases in parotid salivary flow observed in six out of eleven treated subjects and durable transgene expression confirmed at three years post-treatment [198,200]. More recently, aquaporin gene therapy has been extended to Sjögren’s syndrome models, where AAV-mediated AQP1 expression restored fluid secretion and decreased the proinflammatory immune response, broadening the potential clinical applications of this approach [259].

Recent advances in salivary gland organoid systems provide a powerful ex vivo platform to interrogate epithelial ion transport in a tissue-like context. Long-term murine and human major salivary gland organoids can be generated that retain acinar/ductal cellular heterogeneity and exhibit functional responses to neurotransmitter stimulation (e.g., ATP or carbachol-evoked Ca^2+^ responses) [260]. Importantly, organoids also enable quantitative functional assays of transepithelial anion/fluid transport, including CFTR-dependent luminal swelling triggered by forskolin and inhibited by CFTRinh-172, and swelling responses to carbachol stimulation [261]. These approaches create opportunities for the mechanistic dissection of channel coupling (SOCE–ANO1, CFTR-dependent processes) and for patient-relevant drug testing in xerostomia contexts [262].

Despite substantial progress, several important questions remain unresolved. The molecular mechanisms governing direct channel–channel interactions, such as the membrane-delimited inhibition of BK channels by IK activation [164,165], await structural clarification using cryo-electron microscopy and proximity labeling approaches. Whether ion channel expression profiles differ sufficiently between major gland types (parotid, submandibular, sublingual) to explain their distinct secretory properties remains incompletely characterized. The contribution of recently identified channels, including LRRC8 volume-regulated anion channels [134,135,136,137,142,263,264] and additional TRP family members, to physiological secretion requires further genetic validation using conditional knockout models. The molecular identity of the β-adrenergic-stimulated chloride efflux pathway, which is independent of Tmem16A, CFTR, and ClC-2, represents a critical knowledge gap with direct therapeutic implications, as pharmacological activation of this pathway could potentially bypass the calcium signaling defects characteristic of both Sjögren’s syndrome and radiation injury [24]. Additionally, the molecular basis of channel trafficking and polarized membrane targeting, particularly the mechanisms underlying AQP5 mislocalization from apical to basolateral membranes in Sjögren’s syndrome [185,186,187,190], remains poorly understood despite its clear functional significance.

A notable strength of this review is the synthesis of data from genetic ablation studies, electrophysiological recordings, and disease models into a unified framework that reveals organizing principles not apparent from studies of individual channel families in isolation. However, certain limitations should be acknowledged. Much of the available evidence derives from rodent models, particularly mouse knockout studies, and species-specific differences in ion channel expression and function, such as the presence of Kir2.1 in bovine but not in rodent salivary acinar cells [167], may limit direct extrapolation to human physiology. Furthermore, many mechanistic studies have focused on parotid and submandibular glands, with comparatively less known about ion channel integration in sublingual and minor salivary glands. The relative contributions of paracellular versus transcellular transport pathways, and how tight junction composition dynamically regulates paracellular ion flux during active secretion, also remain to be fully elucidated [32].

Future investigations should prioritize several key areas: characterizing ion channel function in human salivary gland organoids and primary cell cultures to bridge the gap between rodent models and clinical physiology [8]; determining whether ion channel-targeted therapies can restore function in established disease rather than merely preventing initial damage; exploring the therapeutic potential of combinatorial strategies that simultaneously target multiple nodes within the secretory network to overcome the functional redundancy that limits single-target pharmacological approaches [155]; and advancing next-generation gene therapy platforms, including AAV-based vectors and ultrasound-assisted nonviral gene transfer, which would enable the repeated administration of AQP1 or other therapeutic transgenes for durable clinical benefit [206,207].

## 10. Conclusions

This review establishes ion channel integration as the fundamental organizing principle of salivary gland fluid secretion. The molecular dissection of individual channel families supports an organized framework in which calcium signaling, chloride conductance, potassium-mediated membrane hyperpolarization, and aquaporin-facilitated water transport operate as functionally coupled modules to generate saliva. The evidence assembled in this review leads to several key insights regarding the organization of salivary gland fluid secretion.

First, the secretory apparatus is organized into functionally ordered steps with distinct rate-limiting control points. Genetic ablation studies consistently identify ANO1-mediated chloride conductance as the indispensable constraint on acinar fluid output, whereas upstream calcium entry pathways and downstream water channels, though essential for maximal secretory capacity, allow for partial compensation when disrupted. This organizational logic has direct therapeutic implications: interventions targeting ANO1 activation may exert greater functional impact than those aimed at more redundant components.

Second, functional redundancy represents a conserved design principle rather than molecular excess. The interchangeability of KCa1.1 and KCa3.1 in maintaining electrochemical driving force, the multiple calcium entry pathways converging on cytosolic calcium elevation, and the parallel chloride uptake mechanisms at the basolateral membrane collectively ensure secretory robustness against single-gene perturbations. This redundancy complicates single-target pharmacological approaches but also suggests that secretory failure in disease states is likely to involve multi-channel dysfunction.

Third, ion channels mediate functions beyond ion conduction. TRPM2 operates as a cellular damage sensor linking oxidative stress to calcium-mediated cell death. Piezo1 transduces mechanical signals during glandular morphogenesis and tissue remodeling. AQP5 participates in regulatory volume decrease through functional coupling with TRPV4. These non-canonical roles expand the potential points of therapeutic intervention and underscore that ion channels integrate diverse cellular inputs beyond their classical electrophysiological functions.

Fourth, channel localization is as critical as channel expression. The striking AQP5 mislocalization observed in Sjögren’s syndrome, where the channel remains expressed but redistributes from apical to basolateral membranes, demonstrates that polarized trafficking determines functional output. Similarly, the apical concentration of IP_3_ receptors ensures that calcium signals initiate at the secretory pole, shaping the spatial pattern of downstream effector activation. Disruption of scaffolding proteins, cytoskeletal elements, or trafficking machinery may therefore produce secretory dysfunction even when channel protein levels remain normal.

Several questions remain unresolved. The molecular mechanisms governing channel–channel interactions, such as the membrane-delimited inhibition of BK channels by IK activation, await structural clarification. Whether ion channel expression profiles differ between major gland types (parotid, submandibular, sublingual) sufficiently explain their distinct secretory properties remains incompletely characterized. The contribution of recently identified channels, including LRRC8 volume-regulated anion channels and additional TRP family members, to physiological secretion requires further genetic validation. Finally, whether ion channel-targeted therapies can restore function in established disease, rather than merely preventing initial damage, represents the critical translational question for conditions such as radiation-induced xerostomia and Sjögren’s syndrome.

Ion channels are increasingly recognized not as isolated conductance pathways but as dynamic integrators of intracellular signaling, epithelial polarity, and transepithelial transport. This integrated perspective offers the mechanistic foundation necessary for developing targeted interventions to restore salivary function in xerostomia arising from radiation therapy, autoimmune disease, aging, or pharmacological side effects. Future studies addressing the regulation of ion channel trafficking, the molecular basis of channel–channel interactions, and the potential for channel-targeted gene therapy will further advance our capacity to treat salivary gland disorders.

## Figures and Tables

**Table 1:** Plasma membrane calcium channels driving salivary gland physiology and pathophysiology.

Channel	Localization	Activation Mechanism	Function in Salivary Glands	Disease Relevance	Ref.
Orai1	Basolateral membrane (near tight junctions)	Store depletion via STIM1 interaction; Ca^2+^-dependent inactivation via calmodulin	Primary SOCE channel; essential for sustained Ca^2+^ influx and fluid secretion	Orai1^−^/^−^ mice: significant loss of SOCE (~90% reduction); required for pilocarpine-stimulated saliva flow; altered agonist-induced Ca^2+^ signals	[33,34,35,36,75,76,77,78]
TRPC1	Basolateral membrane	Store-dependent (STIM1 interaction) and receptor-dependent; may form heteromers with Orai1	Contributes to SOCE; may predominate during weak/oscillatory stimulation; potential scaffold for signaling complexes	TRPC1^−^/^−^ mice: altered agonist-induced Ca^2+^ signals; reduced saliva secretion	[79,80,81,82]
TRPC3	Basolateral membrane	Direct activation by DAG (independent of PKC); can also be store-dependent via STIM1	Receptor-operated Ca^2+^ entry; contributes to both initial Ca^2+^ spike and sustained plateau phases; may form heteromers with TRPC1	TRPC3^−^/^−^ mice: moderate impairment of agonist-induced Ca^2+^ signals and saliva secretion	[83,84,85,86,87]
TRPM2	Plasma membrane (acinar cells)	ADPR binding (generated by PARP during oxidative stress); facilitated by intracellular Ca^2+^	ROS sensor; mediates radiation-induced Ca^2+^ overload, mitochondrial dysfunction, caspase-3 activation, and STIM1 cleavage	TRPM2^−^/^−^ mice: significant protection against radiation-induced salivary gland dysfunction; recovery to ~60–70% of baseline secretion by 30–60 days post-radiation	[46,50,51,52,88]
TRPV4	Basolateral membrane	Hypotonic swelling; mechanical stimuli; moderate heat; arachidonic acid metabolites; synthetic agonists (GSK1016790A, 4α-PDD)	Regulatory volume decrease (RVD); functional coupling with AQP5; contributes to muscarinic-stimulated fluid secretion; integrates thermal and cholinergic signals	TRPV4^−^/^−^ mice: greatly reduced Ca^2+^ entry and impaired RVD in response to hypotonicity; reduced muscarinic-stimulated saliva secretion; impaired temperature-dependent modulation of salivation	[54,55,56,57]
TRPV1	Myoepithelial, acinar, and ductal cells; sensory nerve endings in oral mucosa	Capsaicin; heat (>42 °C); protons; endogenous ligands (anandamide, lipoxygenase products)	Modulates salivation primarily via sensory nerve activation and reflex mechanisms; potentiates carbachol-induced secretion in perfused glands	Pharmacological activation (capsaicin 1 μM): significantly increased carbachol-induced salivation in perfused submandibular glands. Human studies: mouth rinsing with capsaicin or piperine stimulates whole mouth salivary flow	[58,59,60,61,83,89]
TRPM8	Salivary glands (myoepithelial, acinar, ductal cells); trigeminal sensory fibers	Cold temperatures (<26 °C); menthol; synthetic agonist WS-12	Temperature-dependent modulation of salivation; complex context-dependent effects	Pharmacological activation: menthol mouth rinse increases whole mouth saliva flow and protein secretion in humans; however, WS-12 application to perfused glands decreased carbachol-induced salivation, indicating opposing direct vs. reflex-mediated effects	[58,59,63]
L-type VDCCs (CaV1.1, CaV1.2, CaV1.3)	Peripheral cell layers of developing epithelial buds (>50% immunoreactivity in outermost 3 cell layers); minimal in mature acinar cells	Membrane depolarization; sensitive to dihydropyridine blockers (nifedipine)	Branching morphogenesis during development; localized epithelial proliferation via calmodulin-dependent Ras/MAPK/ERK signaling	Pharmacological inhibition (nifedipine): dose-dependent inhibition of epithelial bud formation and cleft initiation in embryonic submandibular gland cultures; ~29% reduction in pERK levels; effects occur in isolated epithelium (not mesenchyme-dependent). Similar inhibition observed in developing lung cultures	[71,72,73,74,90]
Piezo1	Acinar-forming epithelial cells at E14-16 (developmental); upregulated in irradiated tissue	Membrane stretch; membrane tension; mechanical forces (compression, shear stress)	Developmental morphogenesis of secretory epithelia; mechanotransduction; potential role in radiation injury progression	siRNA knockdown: significantly impaired submandibular gland development in organ culture. Radiation injury: PIEZO1 upregulation at gene and protein levels correlates with elevated inflammatory markers and fibrotic markers; proposed as predictive tissue biomarker for xerostomia.	[64,65,66,67,68,69,70,91,92,93]

**Table 2:** Chloride channels mediating transepithelial ion transport in salivary glands.

Channel	Localization	Activation Mechanism	Function in Salivary Glands	Disease Relevance	Ref.
ANO1 (TMEM16A)	Apical membrane of acinar cells	Direct Ca^2+^ binding to transmembrane region; voltage-dependent gating modulated by Ca^2+^ concentration	Primary CaCC mediating rate-limiting Cl^−^ efflux for fluid secretion; transduces muscarinic Ca^2+^ signals into chloride conductance; forms complexes with ERM proteins	ANO1^−^/^−^ mice: perinatal lethality; complete loss of carbachol-stimulated Cl^−^ efflux in acinar cells despite normal Ca^2+^ signaling; establishes it as essential for salivary fluid secretion.	[12,117,118,119,120,121,143,144,145,146,147]
BEST2 (Bestrophin-2)	Acinar cells	Ca^2+^-dependent gating; pentameric channel with unique “neck” and “aperture” architecture	Initially proposed CaCC candidate; generates Ca^2+^-activated Cl^−^ currents in heterologous systems	Best2^−^/^−^ mice: normal carbachol-stimulated Cl^−^ efflux; unimpaired pilocarpine-stimulated saliva production.	[12,148,149,150]
CFTR	Luminal membranes of ductal cells	cAMP/PKA-dependent phosphorylation; ATP binding and hydrolysis; forms complexes with ERM proteins and NHERF1	Ductal Cl^−^ reabsorption; HCO_3_^−^ secretion for pH regulation; coordinates with ENaC for salt reabsorption	CFTR^−^/^−^ mice: stimulated saliva secretion preserved. Human CF patients: elevated Na^+^ and Cl^−^ in saliva confirming role in ductal ion reabsorption.	[21,122,123,124,125,126,127,128,129,151]
LRRC8A/VRAC (SWELL1)	Plasma membrane of acinar cells/ductal cells	Hypotonic cell swelling; heterohexameric assembly with LRRC8B-E subunits; activated within minutes of osmotic challenge	Regulatory volume decrease (RVD); Cl^−^ and organic osmolyte (taurine) efflux; potential ATP release pathway for autocrine/paracrine signaling	Volume-sensitive Cl^−^ currents documented in acinar cells with characteristic VRAC properties. Direct genetic studies of LRRC8 in salivary glands pending.	[134,135,136,137,138,139,140,141,142,152]
ClC-2 (CLCN2)	Basolateral membrane of ductal cells	Hyperpolarization; elevated intracellular Cl^−^; inwardly rectifying	Proposed role in ductal Cl^−^ uptake based on localization and biophysical properties	Clcn2^−^/^−^ mice: complete elimination of hyperpolarization-activated Cl^−^ currents in ductal cells; however, normal pilocarpine-stimulated saliva secretion with unchanged volume, ionic composition, and protein content.	[130,131,132,133,153]
ClC-3 (CLCN3)	Parotid and submandibular glands	Functions primarily as intracellular H^+^/Cl^−^ antiporter in endosomal/synaptic vesicles; proposed but not confirmed plasma membrane function	Vesicular acidification: proposed volume-sensitive Cl^−^ channel (not confirmed); potential indirect effects on glandular blood flow via vascular smooth muscle	Clcn3^−^/^−^ mice: normal swelling-activated Cl^−^ currents; normal Ca^2+^-activated Cl^−^ currents; normal RVD; normal pilocarpine-stimulated saliva secretion with unchanged volume, ionic composition, and protein content.	[130,132,154]

**Table 3:** Potassium and sodium channels regulating salivary gland secretion and ductal modification.

Channel	Localization	Activation Mechanism	Function in Salivary Glands	Disease Relevance	Ref.
KCa1.1 (BK, MaxiK, Slo1)	Basolateral membrane of acinar cells; apical membrane of striated and excretory duct cells	Dual activation: membrane depolarization + elevated intracellular Ca^2+^; Ca^2+^ binding to calcium bowl and RCK1 domain in C-terminus.	Membrane hyperpolarization maintaining driving force for Cl^−^ secretion; regulatory volume decrease (RVD); ductal K^+^ secretion (flow rate-dependent)	KCa1.1^−^/^−^ mice: normal salivary secretion; complete loss of large-conductance K^+^ currents; substantially impaired RVD. KCa1.1^−^/^−^/KCa3.1^−^/^−^ double KO: 65% reduction in fluid secretion; failure to hyperpolarize in response to muscarinic stimulation.	[25,156,158,159,160,161,173]
KCa3.1 (IK1, SK4)	Basolateral membrane of acinar cells; intercalated and striated duct cells	Voltage-independent; activated solely by Ca^2+^ binding to constitutively associated calmodulin at C-terminal domain; EC_50_ ~350 nM Ca^2+^ in parotid acinar cells	Membrane hyperpolarization maintaining driving force for Cl^−^ secretion; functionally redundant with BK channels; may directly inhibit BK channels through membrane-delimited mechanism.	KCa3.1^−^/^−^ mice: normal salivary secretion; normal membrane hyperpolarization to muscarinic stimulation. IK activation inhibits BK channels via physical proximity; N-terminus peptide blocks BK pore; inhibition persists in excised membrane patches.	[25,155,156,161,162,163,164,165,174]
Kir2.1 (KCNJ2)	Basolateral membrane (periphery of acinar cells); interstitial duct segment cells	Inward rectification due to voltage-dependent block by intracellular polyamines and Mg^2+^; conducts K^+^ preferentially into cells at negative potentials	Stabilization of resting membrane potential; K^+^ recycling across basolateral membrane; may specifically support spontaneous secretion in ruminants	Present in bovine parotid but absent in rodent salivary acinar cells (species-specific).	[167,175,176,177,178,179]
ENaC (α, β, γ subunits)	Apical membrane of duct cells	Constitutively active; highly Na^+^-selective; regulated by aldosterone/SGK1, glucocorticoids; negative feedback by intracellular Na^+^ via Nedd4-mediated internalization	Rate-limiting step for ductal Na^+^ reabsorption; generates hypotonic final saliva; electrogenic entry creates lumen-negative potential driving K^+^ secretion; functionally coupled to CFTR	Abolished transepithelial potential difference in perfused rat submandibular duct. Hydrocortisone increases amiloride-sensitive current (blocked by RU486). ENaC and CFTR blockade both impair Na^+^ and Cl^−^ reabsorption (functional coupling).	[8,13,151,157,168,169,170,171,172,180,181,182]

**Table 4:** Aquaporins mediating water transport in salivary gland fluid secretion.

Channel	Localization	Permeability Properties	Function in Salivary Glands	Disease Relevance	Ref.
AQP5	Apical membrane of acinar cells	Orthodox aquaporin; high water selectivity; forms homotetramers with six transmembrane domains per subunit	Primary apical water channel for transcellular fluid secretion; exits water following osmotic gradient established by Cl^−^/Na^+^ secretion	AQP5^−^/^−^ mice: 60% reduction in pilocarpine-stimulated parotid saliva volume; normal gland morphology. Sjögren’s syndrome: apical-to-basolateral mislocalization. Radiation: transcriptional downregulation, increased degradation, disrupted trafficking via ezrin loss.	[12,14,30,31,56,183,184,185,186,187,188,189,190]
AQP3	Basolateral membrane of acinar cells	Aquaglyceroporin; permeable to water, glycerol, urea, and small uncharged solutes; broader selectivity than orthodox aquaporins	Principal basolateral water entry channel; enables water uptake from interstitium to replenish apical secretion	Acinar cells secrete ~1 cell volume/min during stimulation; simple diffusion (Pf ~2–5 × 10^−3^ cm/s) insufficient without massive osmotic gradients compromising viability.	[31,191,192,193,194]
AQP1	Myoepithelial cells; vascular endothelium; absent/minimal in normal acinar and ductal epithelia	Orthodox aquaporin; high water selectivity; prototypical water channel.	Volume homeostasis in myoepithelial cells during contraction; gene therapy target for radiation-induced xerostomia	Gene therapy: adenoviral AQP1 transfer to irradiated parotid glands increased stimulated salivary flow; subjective xerostomia improvement.	[31,195,196,198,199,200,203,204,205,206,207]
AQP4	Minimal in acinar cells; may localize to ductal epithelia and myoepithelial cells	Orthodox aquaporin; high water selectivity	Potential role in ductal or myoepithelial function	Functional contributions to salivary secretion remain unclear; knockout studies in salivary glands not reported	[31,188,191]

**Table 5:** Cotransporters and exchangers sustaining electrolyte gradients in salivary gland secretion.

Transporter/Exchanger	Localization	Transport Mechanism	Function in Salivary Glands	Disease Relevance	Ref.
Na^+^/K^+^-ATPase	Basolateral membrane of acinar and ductal cells	Primary active transport; ATP-dependent exchange of 3 Na^+^ (out) for 2 K^+^ (in)	Generates Na^+^ gradient driving all secondary transport; establishes negative membrane potential; activity increases with parasympathetic stimulation.	Ouabain (Na^+^/K^+^-ATPase inhibitor): 95.8% reduction in ACh-stimulated saliva volume; alteration in salivary cation composition. Muscarinic stimulation increases pump turnover.	[21,208,218,219,220,221]
NKCC1 (SLC12A2)	Basolateral membrane of acinar cells; not expressed in ductal cells	Secondary active cotransport; electroneutral uptake of 1 Na^+^, 1 K^+^, 2 Cl^−^; driven by Na^+^ gradient	Principal Cl^−^ uptake mechanism; accumulates intracellular Cl^−^ ~5-fold above equilibrium; part of “Cl^−^ pump” with Na^+^/K^+^-ATPase and K^+^ channels.	NKCC1^−^/^−^ mice: >60% reduction in muscarinic-stimulated saliva volume; complete loss of bumetanide-sensitive Cl^−^ influx. Human NKCC1 deficiency: severe xerostomia, deafness.	[15,21,156,209,210]
NHE1 (SLC9A1)	Basolateral membrane of acinar and ductal cells	Electroneutral exchange of 1 Na^+^ (in) for 1 H^+^ (out); driven by Na^+^ gradient	Primary pH regulator in acinar cells; couples with Cl^−^/HCO_3_^−^ exchangers for secondary Cl^−^ uptake	NHE1^−^/^−^ mice: complete failure of pHi recovery from acid load in parotid acinar cells. NHE2^−^/^−^ and NHE3^−^/^−^ mice: normal acid extrusion in acinar cells.	[21,211,212,222]
Ae2 (SLC4A2)	Basolateral membrane of acinar cells	Electroneutral exchange of 1 Cl^−^ (in) for 1 HCO_3_^−^ (out)	Secondary Cl^−^ uptake coupled with NHE1; net NaCl uptake pathway	Ae2^−^/^−^ acinar cells: reduced HCO_3_^−^-dependent Cl^−^ uptake. Double Ae4/Ae2 KO: nearly abolished Cl^−^/HCO_3_^−^ exchange.	[214,216,223]
Ae4 (SLC4A9)	Basolateral membrane of acinar cells	Electroneutral Cl^−^/HCO_3_^−^ exchange; activated by cAMP/PKA phosphorylation	Critical Cl^−^ uptake pathway; specifically important for β-adrenergic-stimulated secretion.	Ae4^−^/^−^ mice: 35% reduction in muscarinic + β-adrenergic stimulated salivation; reduced Cl^−^ uptake during cAMP signaling; Ca^2+^-dependent uptake unaffected.	[216,217,223]
SLC26A6 (PAT1)	Apical membrane of ductal cells	Cl^−^/HCO_3_^−^ exchange in secretory direction; couples with CFTR	Luminal HCO_3_^−^ secretion; Cl^−^ reabsorption from ductal fluid; critical for ductal modification	Coupling with CFTR essential for HCO_3_^−^ secretion; severe defect in CF patients and CFTR KO mice. Muscarinic activation enhances Cl^−^/HCO_3_^−^ exchange (acetazolamide-sensitive, Ca^2+^-dependent).	[21,224]

## Data Availability

No new data were created or analyzed in this study.

## Abbreviations

**Abbreviation**	**Definition**
ACh	Acetylcholine
ADPR	ADP-ribose
AE	Anion exchanger
ANO1	Anoctamin-1
AQP	Aquaporin
BK	Big conductance potassium channel
cAMP	Cyclic adenosine monophosphate
CFTR	Cystic fibrosis transmembrane conductance regulator
DAG	Diacylglycerol
ENaC	Epithelial sodium channel
ER	Endoplasmic reticulum
ERK	Extracellular signal-regulated kinase
GPCR	G protein-coupled receptor
IK	Intermediate conductance potassium channel
IP3	Inositol 1,4,5-trisphosphate
IP3R	Inositol 1,4,5-trisphosphate receptor
KCC	K^+^-Cl^−^ cotransporter
LRRC8	Leucine-rich repeat containing 8
M1	Muscarinic receptor type 1
M3	Muscarinic receptor type 3
NBC	Na^+^-HCO_3_^−^ cotransporter
NHE	Na^+^/H^+^ exchanger
NKCC1	Na^+^-K^+^-2Cl^−^ cotransporter
Orai1	Calcium release-activated calcium channel protein 1
PKA	Protein kinase A
PKC	Protein kinase C
PLCβ	Phospholipase C beta
RVD	Regulatory volume decrease
SERCA	Sarco/endoplasmic reticulum Ca^2+^-ATPase
SK	Small conductance potassium channel
SOCE	Store-operated calcium entry
STIM1	Stromal interaction molecule 1
TMEM16A	Transmembrane protein 16A
TRP	Transient receptor potential
TRPC	Transient receptor potential canonical
VRAC	Volume-regulated anion channel
